# Structural and Functional Modulation of Perineuronal Nets: In Search of Important Players with Highlight on Tenascins

**DOI:** 10.3390/cells10061345

**Published:** 2021-05-29

**Authors:** Ana Jakovljević, Milena Tucić, Michaela Blažiková, Andrej Korenić, Yannis Missirlis, Vera Stamenković, Pavle Andjus

**Affiliations:** 1 Center for Laser Microscopy, Institute for Physiology and Biochemistry “Jean Giaja”, Faculty of Biology, University of Belgrade, 11000 Belgrade, Serbia; milena.tucic@bio.bg.ac.rs (M.T.); andrej.korenic@bio.bg.ac.rs (A.K.); or verasekeljic@gmail.com (V.S.); pandjus@bio.bg.ac.rs (P.A.); 2 Light Microscopy Core Facility, Institute of Molecular Genetics CAS, Prague 142 20, Czech Republic; michaela.blazikova@img.cas.cz; 3 Laboratory of Biomechanics and Biomedical Engineering, Department of Mechanical Engineering and Aeronautics, University of Patras, 26504 Patras, Greece; yfmissirlis@gmail.com; 4 Center for Integrative Brain Research, Seattle Children’s Research Institute, 1900 9th Ave, Seattle, WA 98125, USA

**Keywords:** extracellular matrix, perineuronal nets, tenascin-C, synaptic plasticity, mechanotransduction, neurogenesis, super-resolution microscopy

## Abstract

The extracellular matrix (ECM) of the brain plays a crucial role in providing optimal conditions for neuronal function. Interactions between neurons and a specialized form of ECM, perineuronal nets (PNN), are considered a key mechanism for the regulation of brain plasticity. Such an assembly of interconnected structural and regulatory molecules has a prominent role in the control of synaptic plasticity. In this review, we discuss novel ways of studying the interplay between PNN and its regulatory components, particularly tenascins, in the processes of synaptic plasticity, mechanotransduction, and neurogenesis. Since enhanced neuronal activity promotes PNN degradation, it is possible to study PNN remodeling as a dynamical change in the expression and organization of its constituents that is reflected in its ultrastructure. The discovery of these subtle modifications is enabled by the development of super-resolution microscopy and advanced methods of image analysis.

## 1. Introduction

The extracellular matrix (ECM) has an instrumental role in the regulation of function and homeostasis of the nervous system. ECM can exist in a form of condensed web around the neuron and its proximal dendrites, which was first recognized in 1898 by Camillo Golgi in the cat cortex. He named those specific reticular structures perineuronal nets (PNN) [1]. Besides mammals, PNN were also seen in the brains of amphibia, reptiles and birds, and their composition started to unravel only some 30 years ago [2,3].

Initially assumed to function as a rigid mechanical anchor for the cells, PNN were later recognized as long-lasting structures that restrict plasticity and set neuronal circuits’ connectivity. This made them eligible candidates for the regulation of postnatal experience-dependent maturation of brain circuits. After being extensively studied over recent years, it is now clear that the PNN have an active role in signal transduction and act as a reservoir of enzymes, growth factors, immunomodulatory cytokines and chemokines. In this review, we discuss novel ways of studying the interplay between PNN and their regulatory components, taking into account the growing body of literature regarding the relationship between prominent plasticity regulators, through their developmental, behavioral and functional correlations. In addition to PNN’s effect on synaptic plasticity and its structural and functional aspects, in this review we will also cover: (1) the mechanobiological properties of the PNN, such as stiffness, and its influence on cell proliferation, migration, survival and differentiation; (2) the processes of mechanotransduction; and (3) adult neurogenesis. Pointing toward diverse and important functions of PNN, their role in the complex, nonlinear system of the brain with non-equilibrium dynamics, gains in importance for maintaining the homeostasis or allostasis in the face of environmental fluctuations and challenges. We have also dedicated a section of this review to the super-resolution imaging methods as indispensable tools for deciphering the complete topography of PNN and to follow their ultra-structural changes in physiological and pathological conditions.

## 2. Molecular Organization of PNN Structure and Function

Diverse functions of PNN have been described on the molecular, cellular and behavioral levels. Their most prominent role is the control of synaptic formation and its stabilization, which influences the excitation/inhibition balance and the stabilization of neuronal circuits [4,5,6]. Additionally, they act as a buffering system for cations (Ca^2+^ and K^+^) [7], hence increasing the excitability of the interneurons they enwrap [8]. Protective roles of PNN are also shown in Alzheimer’s disease, where they restrict neurons from internalizing Tau proteins, and in the protection from oxidative stress through the accumulation of Fe^2+^ ions [9,10]. Constituents of the PNN (e.g. chondroitin sulfate) can elicit protection in an acute in vitro ischemia/reoxygenation model [11], while PNN are found to limit the effect of genetically impaired antioxidant systems and/or excessive reactive oxygen species production [12]. They can also have modulatory actions on neighboring neurons by scavenging and binding redox-active ions, thus reducing the local oxidative potential (reviewed in [13]). PNN dysfunction is reported in schizophrenia, drug-associated behavior and memory loss, thus pointing to a possible target for potential therapy of neuropsychiatric disorders and cognitive decline [14,15,16,17]. In addition, interplay and coordination of redox interactions with endogenous and exogenous antioxidant defense systems is an emerging area of research interest in anticancer and antidegenerative therapeutics [18].

**Figure 1:**
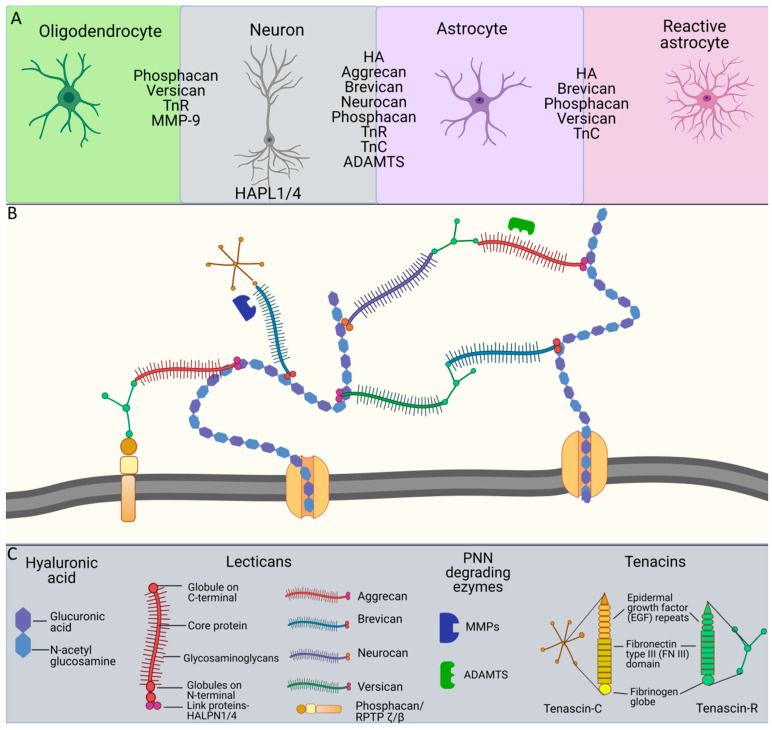
Scheme of perineuronal net structure and assembly. (**A**) Different cell types that are producing PNN components and associated regulatory molecules. (**B**) Schematic representation of the neuronal cell surface depicting a section of the simplified perineuronal net structure and its assembly, showing constituents and associated molecules. (**C**) Graphical representation of various molecular structures used in the above scheme. HA—hyaluronic acid; MMPs—matrix metalloproteinases; ADAMTS—a disintegrin and metalloproteinase with thrombospondin motifs; TA—tenascin assembly; EGF—epidermal growth factor; FN III—fibronectin type III (Created with Biorender.com).

PNN are a mesh of interconnected ECM molecules, secreted by both neurons [19] and glial cells [7], mainly, but not exclusively, enwrapping inhibitory, fast-spiking, parvalbumin expressing (PV^+^) interneurons in the cortex and the hippocampus [20]. These molecules associated with PNN structure and function are hyaluronic acid (HA), chondroitin sulfate proteoglycans (CSPG; including four members of the lectican family: aggrecan, brevican, neurocan and versican), link proteins (cartilage link protein-1-Crtl1; hyaluronan and proteoglycan link protein 1 and 4-HAPLN1/4) and glycoproteins tenascin R (TnR) and tenascin C (TnC) (Figure 1). Every molecule in the PNN contributes to its integrity: HA forms a base to which lecticans bind through connections with link proteins, while TnR interconnects lecticans [21,22,23,24].

HA represents a scaffold of the PNN, synthetized by the transmembrane protein hyaluronan synthase 3 (HAS3), and is composed of unbranched repeated units of glucuronic acid and N-acetylglucosamine [21]. Lecticans consist of globules on their C and N terminals, and of a central core protein with branching repeats of glycosaminoglycan (GAG) made of sulfated glucuronic acid and N-acetylgalactosamine. Lecticans are distinguished by the size of the core protein and the number of GAG chains. The first and still the most common PNN marker is the lectin from the plant *Wisteria floribunda* (WFA, *Wisteria floribunda* agglutinin), which binds to N-acetylgalactosamine residues on the GAG repeats [19,25]. Aggrecan is the most abundant and the most studied lectican in the PNN [26,27]. Within the interplay of neurons and glia, aggrecan and neurocan are primarily synthetized by neurons, while other PNN molecules are expressed in a glia-dependent manner [28]. Investigations of each PNN molecule contribution started on mice with constitutively knocked-out aggrecan, where PNN were not visible, but the synthesis of HA, HAPLN1, brevican and TnR remained unchanged [26]. Later on, animals lacking single and even quadruple PNN components were designed, showing different effects on the synaptic structure and function [26,29,30]. Investigation of PNN in single molecule deficient animals indicated the importance of the lacking molecule in PNN function, however, mice deprived of all four molecules, TnR, TnC, brevican and neurocan, were generated to show the general effects of PNN components deficiency [30]. A new study demonstrated that protein tyrosine phosphatase (RPTPζ), a transmembrane enzyme, and phosphacan as its splicing product, contribute to PNN formation and structure. Mice lacking the gene for RPTPζ lost the net-like structure of PNN, and a new model was proposed, where PNN binds to the neuronal surface not only by HA, but also by RPTPζ [31].

Although PNN are mainly investigated in vivo, there were attempts to investigate the formation of PNN in vitro. One approach was to form PNN-like structures without glial cells, which resulted in the expression of neurocan and phosphacan [32]. In another study, embryonic kidney cells were induced to produce a pericellular matrix resembling the composition of PNN [33]. A condensed pericellular matrix could not be formed without hyaluronan synthase 3, aggrecan, and Crtl1 which binds aggrecan to the HA chain. Due to its complex macromolecular composition and ramified interactions, adequate structure modeling on one hand, and growing PNN in vitro on the other, still represent a challenge.

## 3. Tenascins: Main Properties and Interrelation with Perineuronal Nets

**Figure 2:**
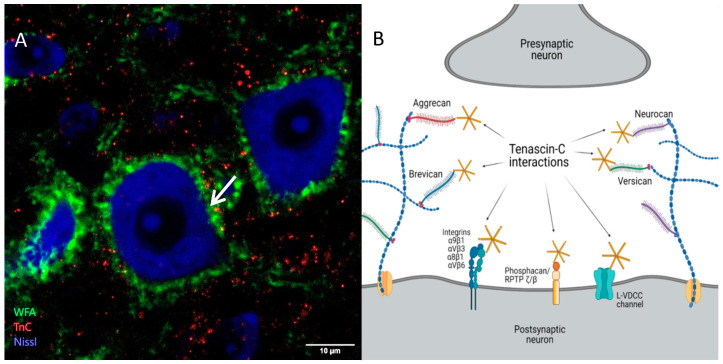
Distribution of TnC and its relation to other ECM components. (**A**) Confocal image of triple staining for PNN (visualized by WFA in green), TnC (red) and neurons (NeuroTrace Nissl stain in blue) in the lateral deep cerebellar nucleus of a 3 month old mouse. Close apposition of PNN and TnC is indicated by the white arrow. (**B**) Expected interactions of TnC with proteoglycans and postsynaptic receptors taking into account their proximity [35,36,37,38,40,41,42,43]. L-VDCC–L type voltage dependent Ca^2+^ channel (Created with Biorender.com).

Besides HA and CSPG, other crucial molecules associated with PNN are members of the tenascin family, TnR and TnC. They differ in structural organization, TnR being a trimer and TnC presenting an assembly of two trimers bound through disulfide bonds to form a hexamer. On their N-terminals, TnC monomers contain the tenascin assembly domain consisting of heptad repeats, followed in sequence by epidermal growth factor (EGF) repeats, fibronectin type III (FN III) domains, and a fibrinogen globe at their C-terminal [34]. Its multidomain structure allows diverse interactions with the surrounding molecules in the ECM and PNN. Namely, TnC has been identified as a ligand for a variety of CSPG [35,36,37,38], and some sites of close proximity of TnC and WFA staining in PNN were observed as well (Figure 2A,B). Due to different binding of molecules to the TnC domains, its function can vary from adhesive to repulsive towards neurons, as developmentally regulated by alternative splicing [39]. In the neural tissue, TnC performs its functions mostly through the direct interaction with different cell surface receptors, including integrins, cell adhesion molecules and phosphacan/RPTP ζ/β [40,41,42,43]. However, TnC can act indirectly as well, through the modulation of other ECM components (Figure 2B).

In addition to structural differences, tenascins also differ in their expression patterns—TnC can be detected in the brain of the developing mouse at E10 [44] while the expression of TnR is more pronounced in adults [45]. In the first few weeks of life, brain ECM consists of TnC, as well as of neurocan and versican and such a structural composition corresponds to a more diffuse state of ECM organization that may allow for the modulation of connectivity in the developing brain. In the course of time, the expression profile of proteoglycans changes towards smaller molecules, such as aggrecan, brevican and TnR (with downregulation of TnC) that can participate in the building of a more condensed ECM and PNN, hence restricting and stabilizing established synaptic connections [46].

TnC is secreted in the extracellular space by astrocytes, radial glial cells and a restricted number of neurons, such as the granule cells (GC) in the dentate gyrus (DG) of the hippocampus, the motor neurons in the spinal cord, or the pyramidal cells in the cortex [44,47]. In the adult brain, TnC expression persists in the hippocampus, cortex, hypothalamus and cerebellum [48,49,50]. However, the expression of TnC can be persistently upregulated under certain pathological conditions, such as the Ammon’s horn sclerosis [51]. The healthy human hippocampus has distinctive boundaries of low and high TnC expression. The layers with major synaptic inputs such as *stratum lacunosum moleculare*, pleomorphic cell layer, GC zone and subgranular cell layer are characterized as areas with high expression of TnC. Contrary to that, in patients with Ammon’s horn sclerosis, as a consequence of reactive astrogliosis, the distribution boundaries of TnC are lost, and it becomes evenly expressed in most hippocampal layers resulting in the pathogenic aberrant axonal reorganization [51].

TnR is proven to be an indispensable structural constituent of PNN, necessary for aggrecan clustering and net stabilization [22]. It has been shown that the expression of brevican, HA and neurocan is down-regulated in mice deficient for TnR [45]. Furthermore, exogenous addition of TnC could not rescue the PNN assembly, implying that the role of TnC is more regulatory than structural [22]. Additionally, the expression of phosphacan and neurocan is diminished in TnR/parvalbumin double knock-out mice [52]. The interaction of brevican and TnR has been shown to be essential in the maintenance of PNN integrity in the midbrain, cerebellum and medulla oblongata [53].

A study on the first established TnC-deficient mouse line showed that, on the molecular level, the absence of TnC in the brain causes a decrease in serotonin and dopamine production in the cerebral cortex, hippocampus and striatum, while on the behavioral level, these mice exhibit hyperlocomotion and poor swimming ability [54]. Another independent research group generated a new constitutive TnC-deficient mouse line [55], with a behavioral profile showing spontaneous nocturnal hyperactivity, poor sensorimotor coordination and low swimming velocity due to reduced muscle strength [56,57]. It has been shown that PNN appears to be normal in the hippocampus of 4-to 6-week-old TnC deficient mice compared to wild type littermates [55]. Furthermore, the expression of TnC in the adult mouse cortex in relation to its possible influence on the structure of PNN surrounding PV^+^ cells was also investigated by Irintchev and colleagues, 2005 [58]. They revealed generally low expression of TnC in the cortical areas containing numerous WFA positive neurons, implying that TnC is not a major component of PNN in the mouse cortex. In addition, WFA labeling revealed a normal appearance of PNN in TnC-deficient mice as compared to wild type littermates. However, Stamenkovic et al. 2017, [50] showed that although the appearance of PNN was normal in the cerebellum of 7-week old TnC-deficient mice, they were less condensed in 11-week-old TnC-deficient mice, which was evidenced by a reduced WFA staining. This finding may suggest a possible region-specific and/or time-dependent role of TnC in maintaining the structural integrity of PNN or an altered developmental maturation and stabilization of PNN in the absence of TnC. Finally, a recent study [30] demonstrated that the maturation of PNN in the CA2 region of the hippocampus was delayed in mice lacking four ECM molecules: TnC, TnR, neurocan and brevican. However, the contribution of individual molecules to the effect was not clearly established. Overall, since the assembly of PNN is not significantly altered by the lack of TnC, it seems that the role of TnC in PNN is rather more regulatory then structural. It still remains to be elucidated whether such an effect could be achieved indirectly through the interaction with lecticans, as with the well-established PNN components.

**Figure 3:**
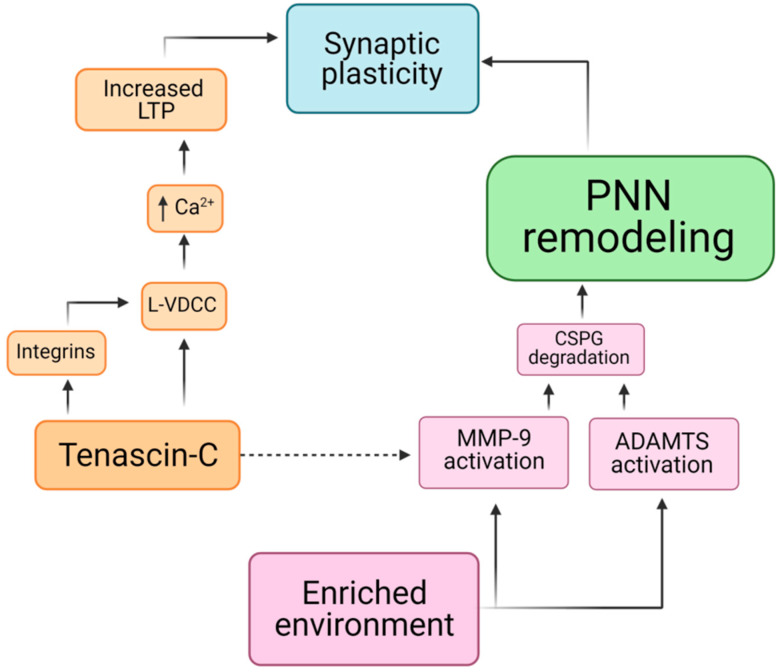
Hypothetical block diagram that illustrates the possible pathways of PNN remodeling towards synaptic plasticity and the role of enriched environment, including the involvement of tenascin C. LTP—long-term potentiation; L-VDCC-L—type voltage dependent Ca^2+^ channel; CSPG—chondroitin sulfate proteoglycans; MMP-9—matrix metalloproteinase 9; ADAMTS—a disintegrin and metalloproteinase with thrombospondin motifs. The dashed line indicates that direct evidence is still lacking (Created in Biorender.com).

On the functional level, TnC is involved in the shaping of neuronal circuitry in the hippocampus, whereas its deficit causes abnormalities in gamma rhythm [59]. These changes in electrophysiological properties may stem from a decreased number of somatostatin-expressing interneurons that are specifically confined to the CA1 region, thus, leaving the gamma rhythm generator found in the DG unaffected. TnC-deficient mice also showed a reduced long-term potentiation (LTP) in the CA1 region of the hippocampus, as a consequence of affected L-type voltage dependent Ca^2+^ channel (L-VDCC) mediated signaling (Figure 3). Thus, it was shown that, by increasing Ca^2+^ influx leading to LTP, TnC affects certain forms of synaptic plasticity involved in the basic mechanisms of learning and memory [55]. A hypothetical mechanism of synaptic plasticity was suggested, either through direct interaction of TnC with L-VDCC, or through a pathway that includes integrins and tyrosine kinase of the Src family [60].

## 4. Functions of Perineuronal Nets in Neuronal Plasticity

During its development, the central nervous system (CNS) is highly sensitive to internal and external stimuli and exhibits increased levels of neuronal plasticity. Neuronal plasticity includes changes in synaptic connections and neurons themselves over time. Substantial changes, such as the outgrowth of new dendritic spines, the formation of new synapses and possibly new neurons, are processes of structural plasticity that underlie long-term memory [61]. Structural neuronal plasticity is described as an anatomical modification of the brain that is correlated with the process of learning, manifested through the occurrence of new synapses (synaptogenesis) and new neurons (neurogenesis), followed by remodeling of ECM [6]. Functional plasticity is most prominently seen after injury, when neurons gain new functions [62]. Activity-driven processes, together with synaptic pruning, are essential for making functional brain circuits during normal brain development. On the other hand, during adulthood, neuronal plasticity is responsible for learning, memory and recovery after injury [63,64].

It has been shown that the brain is most easily modified by experience in the early age, when a peak in neuronal plasticity is defined as the critical period, or the plasticity window [65]. Immunostaining of neurocan and WFA in the rat visual cortex (V1) demonstrated that the critical period of the early development closes with the formation of PNN and stabilization of existing synapses occurs around the third postnatal week [66]. On the other hand, it was shown by novel object recognition tests, that the rat hippocampus enters a critical period which is completed around postnatal day 17 (P17) [67]. In the rat medial entorhinal cortex, a visible formation of PNN commences at P12, already gaining their complete form at P17 [68]. The appearance of molecular and behavioral markers of the critical period ending may show slight temporal differences among different brain areas, also possibly depending on the environment.

The property of PNN to facilitate proper neuronal connections at an early age is preserved throughout the lifetime since the net is in a loose state in the first couple of postnatal days, gradually condensing with age [65]. Therefore, the main protective role of PNN, in terms of plasticity, is reflected as suppressive [6]. However, since PNN are dynamic structures regulated in an activity-dependent manner, their enzymatic degradation may allow a reactivation of neuronal plasticity.

PNN in the cortex and hippocampus mainly condense around PV^+^ interneurons. Among the identified factors regulating both the onset and the closure of the critical period for maturation of the PV^+^ interneurons in the murine visual cortex is Otx2, the embryonic homeoprotein that binds to the PNN [69,70]. PV^+^ neurons receive inputs on their soma from excitatory glutamatergic neurons and as a response they release GABA, thus participating in the synchronization of neural networks. The relative PV expression in GABAergic interneurons in the hippocampus is dependent on the presence or absence of PNN [4]. Namely, intensely stained PV^+^ interneurons are likely to be enwrapped by PNN, while weakly stained PV^+^ interneurons tend to lack PNN. Furthermore, PNN affect cell excitability and excitatory input to the interneurons they surround. That was shown in a recent study in the medial prefrontal cortex, where PV^+^ cells enwrapped with PNN had higher density of perisomatic excitatory and inhibitory puncta and longer axonal initial segments when compared to PV^+^ cells without PNN [5]. When the expression of PV^+^ interneurons in the frontal, motor, somatosensory and visual cortex is reduced by intermittent theta-burst stimulation on the other hand, it was followed by an enlargement of PNN around them, demonstrating that the proper expression of both PV and PNN is crucial for neuronal plasticity [71].

One of the most investigated properties of PNN is their effect on synaptic plasticity. It can be explored functionally, by measuring LTP to determine the synaptic strength between neurons, and structurally, by imaging dendritic spines and protrusions of dendrites that make synapses with axons [72]. Interestingly, the expression of reactive carbohydrate motif on the neuronal surface may precede the location of synaptogenesis. Namely, the expression of antibody Cat315, which binds to RPTPζ in developing rodents and to aggrecan in the adult brain, was detected prior to sites of synaptic formation in the primary cortical culture [73]. Structural remodeling of synapses was shown in a study with in vivo two-photon imaging of dendritic spines in the visual cortex. Namely, after the addition of CSPG-degrading enzyme chondroitinase ABC (chABC), dendritic spines went through structural remodeling, with spine heads becoming larger in the absence of PNN [74].

In aged brains, PNN becomes inhibitory for synaptic plasticity. On the molecular level, this is caused by a decrease in 6-sulphation of their glycan chains (C6S) [75,76]. On the level of synaptic complex, PNN physically limits lateral diffusion of α-amino-3-hydroxy-5-methyl-4-isoxazolepropionic acid (AMPA) receptors, which was observed after enzymatic degradation of PNN around neurons in the rat hippocampal culture [77]. Digesting HA in the rat cultured hippocampal network leads to hyperexcitability, an epileptic form of activity that was suppressed by blocking AMPA and L-type voltage dependent Ca^2+^ channels (L-VDCC), thus supporting the role of PNN constituents in optimizing neuronal networks through the control of synaptic inputs [78]. Interestingly, after enzymatic degradation of CSPG, the effects went beyond only physical remodeling since free chondroitin sulfate chains may even mimic the function of glutamate through the interaction with AMPA and kainate receptors. The effect was shown by voltage and patch clamp recordings on rat hippocampal neurons, where eliciting Na^+^ and Ca^2+^ currents led to cell depolarization [79]. However, an alternative explanation for perineuronal nets control of synaptic plasticity lays in its acting as a physical barrier to incoming synapses due to the binding of the repulsive guiding cue molecule semaphorin3A [80].

One of the main problems in the investigation of PNN function in normal and pathological conditions is the unselective degradation of lecticans. The molecular mechanisms that underlie the PNN role in synaptic plasticity could be fully addressed by selectively digesting PNN constituents such as individual CSPG and HA, as well as by generating knock-out animals for specific glycoproteins, link proteins or lecticans. For example, by using brevican mutants, it was demonstrated that brevican affects hippocampal PV^+^ interneurons by controlling the localization of K^+^ channels and the level of AMPA receptors [29]. On a larger scale, synchronized activity of neurons is changed after the disruption of PNN, setting the brain in a juvenile-like state with a decreased inhibition in an ocular deprivation experiment, while increasing the frequency of sharp wave ripples [81]. These ripples represent a synchronized network activity of pyramidal principal cells and interneurons in the hippocampus, which occurs during resting and slow wave sleep in order to consolidate memory. It is proposed that PNN removal by chondroitinase or hyaluronidase around PV^+^ interneurons decreases the secretion of GABA, leading to an increased excitability of pyramidal cells and destabilization of neuronal network activity [82]. As seen from the above studies, the role of PNN in synaptogenesis may be dual, stabilizing, as well as restrictive. However, PNN are not just static barriers for synapse formation, but their every component individually and collectively acts to actively participate in stabilizing the excitation/inhibition balance, hence justifying the view of the brain’s ECM as a part of the tetrapartite synapse (consisting of presynapse, postsynapse, glia and ECM) [83].

## 5. Remodeling of Perineuronal Nets

Depending on experimental conditions, PNN disruption can be evoked by adding chondroitinase and hyaluronidase, or by activating physiological pathways which include several families of ECM degrading enzymes [50,84]. These enzymes degrade various ECM molecules including CSPG, adhesion molecules and growth factors. Two families of ECM degrading proteins are of particular importance for PNN degradation: matrix metalloproteinases (MMPs) and a disintegrin and metalloproteinase with thrombospondin motifs (ADAMTS). Lectican degrading enzymes found in the hippocampus and neocortex are ADAMTS, especially ADAMTS15 and four that are found to be up-regulated at the end of the second postnatal week, matching the critical period for synaptogenesis [84]. Likewise, an increased activity of MMP-9 was found in the auditory cortex, promoting degradation of the PNN around PV^+^ interneurons and leading to sensory hypersensitivity in the Fragile X syndrome animal model [85]. Additionally, gel zymography revealed an increased activity of MMP-9, but not MMP-2, in the cerebellum of mice housed for 4 weeks in the conditions of elevated neuronal stimulation induced by an enriched environment (EE), in comparison to standard conditions (SC), even though the appearance of PNN was not apparently changed at this time point [50].

However, by prolonging the time of exposure to EE from 4 to 8 weeks, PNN staining was decreased in the cerebellum of wild type mice, while it was even slightly increased in MMP-9 and TnC deficient mice, indicating reduced structural plasticity in the absence of either of these two molecules. Finally, TnC deficient mice housed in SC and EE for 8 weeks were reported to show a decreased activity of MMP-9 regardless of housing conditions, implying that TnC is required to regulate proteolytic degradation of PNN by modulating the activity of MMP-9 [50]. The exact role of TnC in PNN remodeling remains to be further elucidated by means of methods that investigate protein-protein interactions such as super-resolution microscopy techniques or FRET (Förster resonance energy transfer) microscopy.

EE causes a variety of changes in the brain; thus, one month of exposing animals to EE leads to a lower excitation/inhibition density ratio and a lower PV expression in the hippocampus, which corresponds to enhanced structural plasticity and a high memory consolidation state [86]. It was also shown that after the same exposure time to EE, the expression of PV and brevican turned to be down-regulated in the hippocampus, with synaptic modifications observed on PV^+^ neurons [29]. The proposed mechanism for the role of brevican in learning new tasks implies that PV and brevican up-regulation synchronizes pyramidal cells that are responsible for the formation of short-term memory. Indeed, the relationship between PNN remodeling and the processes of learning and memory is orchestrated by the interactions of PNN degrading enzymes, lecticans and ECM glycoproteins (Figure 3).

Considering that both ECM and PNN consist of the same building components, differences in their abundance results in a different molecular organization and function. It should be stressed, however, that the procedures that cause degradation (acute or chronic) of HA and CSPG, affect both ECM and PNN, so the effect should be taken with caution. Nevertheless, based on the known data of the role of PNN in synaptic coverage, we have focused in this review on the documented correlations of their modification and plasticity changes in different tissue structures, albeit caution needs to be exercised if direct causality is sought. 

## 6. Perineuronal Nets and Tenascin-C in Mechanotransduction

As we have already outlined above, the ECM provides conditions for normal cellular functioning including synaptic transmission and plasticity, through building a network that maintains tissue structure allowing the communication of cells with their environment. The structural composition of this network, which provides the support for the shaping and positioning of cells, is maintained through the integration and transmission of the mechanical forces generated within cells towards the whole tissue and *vice versa* [87]. ECM proteins were initially presumed to be creating a rigid mechanical anchor for the cells. It is now familiar that the ECM has an active role in signal transduction and acts as a reservoir of enzymes, growth factors, immunomodulatory cytokines, and chemokines. Moreover, the mechanobiological properties of the ECM, such as stiffness, have an influence on cell proliferation, migration, survival and differentiation.

By using atomic force microscopy, Jiang and colleagues tried to examine how developing neurons adjust their own stiffness in response to the stiffness of the surrounding ECM [88]. They have shown on spinal cord neurons cultured on a polyacrylamide gel, that neurites may respond differently compared to cell soma. Moreover, they noticed that an area surrounding the neural soma had lower stiffness than the hydrogel substrates, and speculated that these extracellular structures present in the culture were PNN. It is thus possible that the cells produce and secrete ECM components, including proteoglycans, as part of a response to mechanical cues.

After brain injury, PNN that usually restrict synaptic plasticity undergo extensive changes in their structure and activity [89]. Traumatic brain injury (TBI) causes a general decrease in CSPG around the injured site, making the environment more growth permissive. Thus, by opening the window of plasticity reparation mechanisms can be activated. Western blot analysis for versican, neurocan and aggrecan showed a decreased protein expression after TBI [90]. Additionally, a decrease in the number of WFA-, aggrecan-, and phosphacan-positive PNN was reported after TBI in the entire injured cortex [91]. Consistent with these results, Vita et al. reported a loss in PNN staining after traumatic brain injury [92]. Moreover, vascular damage and stroke, as well as CNS injury are followed by an increased MMP activity observed in the first couple of days after the injury, which results in a degradation of perivascular and perineuronal ECM, but also of PNN [93]. In fact, ECM of the CNS undergoes the dynamics of continuous turnover, the ECM degrading enzymes regulating this event in an activity-dependent manner. What are the exact regulatory molecular mechanisms of degrading enzymes that direct the local environment towards PNN degradation need to be further investigated, so that the activation of plastic changes can be employed for therapeutic purposes.

TnC belongs to the group of matricellular proteins comprised of non-structural ECM molecules that are highly upregulated during tissue remodeling [94]. It is present in tissues under strong mechanical pressure, such as the places where muscles and bones connect to tendons [95]. TnC is a highly elastic molecule that can be stretched to several times of its resting length [96], thus directly contributing to tissue elasticity and protection from mechanical stress. Although the signal transduction triggered by mechanical stimuli that leads to increased synthesis of TnC is not completely understood, it has been shown that the expression of TnC in fibroblasts is sensitive to mechanical stress [97]. On the other hand, interaction between TnC and integrins, cellular membrane receptors that physically link ECM and the cytoskeleton, has been recognized early on. At least four integrins, α9β1, αVβ3, α8β1 and αVβ6, recognize the FN-III domains of TnC that acts as a ligand [40,41]. Moreover, α9 subunit of integrins promotes neurite outgrowth and an increased axonal regeneration after spinal cord injury in cell cultures grown on TnC [42]. Neurite outgrowth requires cytoskeleton reorganization in order for the neurite formation to be initiated. Downstream signal transduction at the membrane into the cell is carried out through the Rho-mediated signaling pathway that is involved in control of cell morphology, survival, proliferation and adhesion, which are all dependent on matrix stiffness [98]. In addition, it was shown that TnC has an inhibitory effect on cell contractility in Rho-mediated signaling pathways [99].

The scratch wound assay, an in vitro experimental model of mechanical injury, is used for studying cell migration and cell–cell interactions, and to that effect it has been shown that mechanical injury induces boundary astrocytes to produce TnC [100]. This further causes promotion of cell proliferation and migration via the β1 integrin pathway, thus initiating the tissue regeneration by forming a glial scar. In the light of recent knowledge on the regenerative role of the glial scar [101] it was emphasized that its formation helps the healing process by isolating the damaged area. In cancer, TnC and other ECM components undergo an important interplay. Namely, in glioblastoma, aggrecan is down-regulated, while TnC, neurocan, versican and HA are overexpressed [102,103]. Overexpressed TnC binds to lecticans, which increases the overall density and stiffness of ECM in brain cancer [104]. Although the overall metabolism in cancer is altered as compared to healthy tissue, TnC could have clinically relevant role for ECM mechanical properties in cancer.

Mechanical stress that causes chronic pain can result in maladaptation of the CNS. Reorganization of the ECM is necessary for maintenance of the homeostasis. Since the hippocampus has an important role in the experience of pain, hippocampal cellular plasticity provides the mechanisms for extracellular alterations that allow the adaptation to these changes. Atomic force microscopy results have shown that an injury that causes chronic pain is followed by a decreased stiffness of the hippocampal ECM, as well as a decreased dendritic complexity [105]. Moreover, an immunohistochemical analysis has shown a decline only in the number of PV^+^ interneurons enwrapped by PNN, while PV^+^ interneurons without developed PNN were not affected. This complements the understanding that PNN have an important regulatory role in the activity of PV^+^ interneurons.

## 7. Perineuronal Nets and Tenascin-C in Regulation of Neurogenesis

Although the ability of the nervous system to adapt to the environment decreases in adulthood, it still retains significant capabilities for structural remodeling and functional adaptation. The process of adult neurogenesis, which is considered a special type of structural plasticity, takes place in the subgranular zone (SGZ) of the hippocampus and the subventricular zone lining the cerebral ventricles [106]. In these two neurogenic niches new neurons are formed through the differentiation of neural stem cells (NSC), and the entire process is supported by specialized ECM components and the surrounding cells that form a dynamic and highly interactive microenvironment.

In the process of becoming functionally mature, developing cells receive extensive synaptic inputs, while interneurons play a significant role in the regulation of adult neurogenesis [107]. The activity of PV^+^ interneurons is involved in the early phases of adult hippocampal neurogenesis in an experience-dependent manner, as well as in the later stages [108]. Namely, optogenetic activation of PV^+^ neurons promoted cell survival, while suppression of their activity reduced the survival of newborn cells. PV-expressing interneurons provide GABA signalization with two distinct spatiotemporal profiles: they evoke fast postsynaptic currents in mature GC, while in both mature and newborn GC they can elicit slow postsynaptic currents [109]. During synapse maturation slow postsynaptic responses transform into faster ones, while the formation of synapses between newborn GC and GABAergic PV^+^ interneurons is considered to take place around 6–8 weeks [110]. Changes in the number of neurons in the adult DG causes massive synaptic reorganization and alters hippocampal excitability [111]. In addition, the finding that the PNN formation is driven by neuronal activity is supported by the finding that cultured neurons in conditions of suppressed presynaptic transmitter release show a reduced WFA signal [112].

It is possible that the changes in synaptic activity may regulate the expression of PNN. However, besides the association of PNN enwrapping PV^+^ interneurons with the adult hippocampal circuitry, PNN have not been fully investigated in the context of regulation of adult neurogenesis. Immature neurons are considered to be highly plastic due to their increased excitability and enhanced synaptic plasticity in the period of 4–8 weeks after birth [113], so it is not expected for them to be surrounded by PNN. Even after reaching the maturation stage, there is no evidence that PNN enwrap newborn neurons in adulthood, to help synapse stabilization. In vitro studies show that developing cortical neurons are capable to independently synthesize HA [114]. The specific role of HA may depend on neuronal maturation and the presence of other PNN components. The presence of structural components of PNN, HA and Crtl1, triggers the formation of the network [33]. In fact, HEK cells transfected to generate HAS can form a diffuse matrix. However, in the absence of HA in the medium, aggrecan and Crtl1 are dissipated around the cells, but after synthesis of HA by HAS, these two molecules were incorporated into the pericellular matrix. In addition, the matrix around HEK cells showed the same biochemical properties as PNN.

As reviewed by Su et al. 2019, HA has a regulatory effect on neurogenesis both in the developing and the adult brain, through the regulation of NSC expansion and the early stages of neuronal differentiation and maturation, and through the regulation of neuronal activity, respectively [115]. It has been shown that the inhibition of HA-dependent signaling pathways in the hippocampus leads to elevated proliferation rate of NSC. Since HA in PNN forms a backbone to which other molecules bind, it enables the regulation of functions of mature neurons. HA is synthesized by NSC and its expression increases with age in the SGZ, which could be related to a reduction in NSC expansion and differentiation in this zone [116]. The importance for adult neurogenesis of another PNN component, CSPG, has been shown by their pharmacological and genetic depletion [117]. In physiological conditions NSC and neuronal progenitors are covered with CSPG and their depletion results in impaired GC production in the DG and cognitive memory impairment. Interestingly, rearing animals in EE for two weeks accelerates the expression of CSPG and promotes the production of GC. Substantial evidence supports the involvement of CSPG in the regulation of all major stages of neuronal development: cell proliferation, migration and axonal guidance, as well as synapse formation and stabilization [118]. As a constitutive part of the NSC environment, CSPG orchestrates the proliferation and migration of neurogenic and gliogenic stem cells through the regulation of growth factor responsiveness [119]. Studies considering involvement of single PNN components in neurogenesis depend on the context (e.g. the use of pericellular matrix) and do not provide direct evidence of PNN impact on adult neurogenesis. Considering the specific organization of the neurogenic niche, there may be significant differences yet to be elucidated.

TnC, as a prominent constituent of the stem cell niche [120], can associate with CSPG and affect processes of adult neurogenesis. In addition, studies on TnC knock-out mice have shown its important roles in proliferation, migration, differentiation and survival of NSC and the oligodendrocyte precursor cells [121,122]. In a recent work on cultured neurons from animals deficient in four PNN components, tenascin-C, tenascin-R, aggrecan and brevican, an increase in excitatory synaptic components was demonstrated [30]. Future studies regarding the participation of ECM components in the regulation of adult neurogenesis should be able to provide insight into mechanisms guiding this process.

## 8. Perineuronal Nets Visualization Techniques and Ultrastructure Analysis

**Figure 4:**
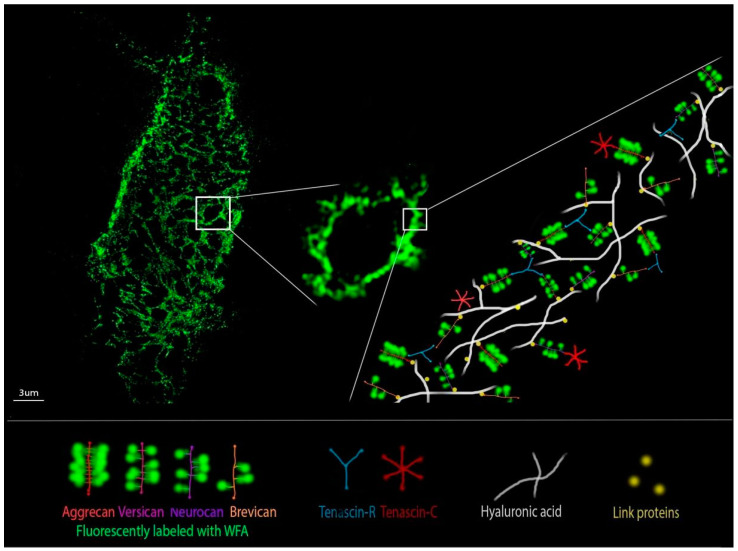
An original SIM image of PNN in the hippocampus (**left**), and an enlarged image window with a schematic representation of the main PNN constituents and associated molecules (enlisted in the bottom) and their simplified relations (**right**). The schematic representation emphasizes that only the lecticans, fluorescently labeled with WFA as the PNN marker, form the observable and analyzable PNN topography.

Although techniques exist to isolate PNN and quantify its GAG [123] the development of advanced light microscopy techniques has been of particular significance for the investigation of PNN structure and function. After discovering the composition of PNN, and their regional brain distribution pattern, PNN were characterized with epifluorescent and confocal microscopy by assessing the intensity of fluorescence staining of their components and by estimating their abundance across tissue layers [7,19,124,125]. Although it was obvious from the first discovery that PNN have unique geometry that consists of an assembly made of polygonal units, mainly pentagons and hexagons, only after the development of super-resolution techniques, it was possible to elucidate it [126,127]. The distribution and structure of PNN are mostly assessed through measurements of WFA signal intensity (Figure 4). Since WFA binds nonspecifically to lecticans, we are often missing the information on the expression of HA, TnR and link proteins required for obtaining the complete picture about the PNN architecture. Known microscopy techniques and labeling approaches are presented in Table 1 and we will illustrate here in some detail techniques that were instrumental in advancing our knowledge of PNN, offering possibilities for further advancements.

A pioneer study on PNN geometry was performed by Arnst and colleagues in 2016, in which they investigated the microstructure of PNN in the somatosensory cortex of rodents [128]. They showed that WFA labeling corresponds to a polygonal mesh with the WFA intensity maxima as vertices. The single mesh in the PNN of rodents could exist in two different patterns of WFA distribution pointing to the necessity of being able to discriminate between even distribution or WFA aggregation that is making vertices. Due to the limitation in spatial resolution, no further detailed analysis was possible at that point. This study was focused on the investigation of the microstructure of PNN in rodents of normal physiology. It revealed the main and the most intriguing characteristic of PNN, the formation of compartments on the cell surface distributing molecules in a dynamically changing pattern that depends on the external stimulus, permeating the physiological or pathological state of the organism.

Since dimensions of structural elements of the PNN organization are near or below the diffraction limit of light microscopy, more efficient analysis was enabled by employing super-resolution microscopy. In the study by Dzyubenko et al, 2018, Structured Illumination Microscopy (SIM) allowed for quantitative analysis of cortical PNN topology [126]. Quantitative analysis of cortical PNN topology was performed in mild hypoperfusion and focal cerebral ischemia in the rat brain. The study combined 3D imaging by SIM with mathematical reconstruction and analysis of PNN mesh vertices. Topological measurements revealed that PNN do not simply go to a degradation state in ischemia, but instead the vertices change their interlinking. Thus, PNN in mild hypoperfusion and ischemia could have been in the specialized tension and dissociation state, possibly returning to original organization after the stroke and thus maintaining the balance between neuroplasticity and neuroprotection.

Using the same microscopy technique and image analysis algorithm according to [126], in order to image PNN in the hippocampus of TnC-deficient and wild-type mice after rearing in EE, we revealed subtle differences between the ultrastructure of cortical and hippocampal PNN. Moreover, results shed light on the effects of genotype and environment contributing to the brain regional differences, assuming that the mechanism of PNN remodeling, caused by the deficiency of TnC, remained the same in all hippocampal regions [129].

In the study by Sigal in 2019 on the mouse model of Rett syndrome, super-resolution Stochastic Optical Reconstruction Microscopy (STORM) was used to quantify the changes that define PNN ultrastructure around cortical PV^+^ interneurons and their perforating synapses [127]. Image analysis was performed on the PNN ultrastructure in WFA^+^/PV^+^ cells in 2D, and a correlation was found between contiguity of PNN ultrastructure and surface intensity, where PNN with stronger WFA signal intensity had smaller meshes. That finding implies that the signal intensity of WFA as a PNN marker follows the complexity of the condensed state. Besides that, for the first time, PNN were imaged in relation to perforating inhibitory and excitatory synapses, revealing a shift in the synaptic balance towards PV^+^ interneurons that led to greater network activity in mature animals.

Both super-resolution studies (SIM and STORM) were able to evaluate specific PNN mesh parameters and point to so far unobserved differences in PNN among animals under different pathological conditions. Both approaches also took the 3D ultrastructure into account, either by determining the PNN topology directly or by using a cylindrical surface projection. Thus, it would be of interest to use both approaches and evaluate more parameters in all images from both SIM and STORM, in order to compare super-resolution imaging methods.

## 9. Conclusions

ECM in the brain plays a crucial role in maintaining cellular homeostasis and in allowing the system to adapt to changes. Neuronal plasticity is the key mechanism that provides proper development and reaction to internal and external stimuli during adulthood, as well as regeneration after mechanical injury. Short-term changes pertain to strengthening already existing synapses, while the long-term changes pertain to the ability to create new neurons. Continuous differentiation of NSC during life implicates functional integration by involving new neurons into existing circuits. New neurons receive both excitatory and inhibitory synaptic inputs from nearby cells, which govern their development. They are also able to send axonal projections to other cells and create new synapses. The massive synapse reorganization is a highly dynamic process and is followed by changes in ECM.

Stabilization of established synapses, as well as the formation of new synapses, is mediated by PNN through various interactions of their constituents with membrane receptors and channels. TnC is involved in fine tuning of the balance between excitation and inhibition; however, its modulatory role in PNN structuring remains to be elucidated. Structural changes in PNN topography, such as the condensation state and interlink pattern, control the synaptic plasticity. Deciphering the complete topography of PNN will require advanced imaging and analysis of all its constituents in physiological and pathological conditions. Super-resolution imaging methods, such as SIM and STORM, along with advanced image analysis techniques, offer tools for addressing the complete role of PNN ultrastructural changes in other physiological and pathological conditions. These methods are invaluable for elucidating the complexity of molecular interactions that give rise to the balance between the plasticity and the consistency of neural networks.

## Figures and Tables

**Table 1:** Chronology of the development of microscopy techniques applied to the investigation of PNN structure with specific labeling markers.

Microscopy Technique	PNN Characteristics	PNN Labeling	Reference
Fluorescent microscopy	Ability to bind WFA	WFA	[19]
Light microscopy	Distribution patterns	WFA, *Vicia villosa* agglutinin, colloidal iron hydroxide staining	[125]
Immunoelectron microscopy	Brevican-TnR colocalization	Brevican TnR	[53]
Confocal laser scanning microscope, electron microscopy	Staining intensity in TnR KO mice	TnR Hyaluronic acid Phosphacan Neurocan Brevican	[45]
Confocal fluorescence microscopy	Morphological alteration in TnR/PV KO mice	Phosphacan Neurocan WFA	[52]
Nuclear microscopy (iPIXE)	Accumulation of metal ions	Colloidal iron	[10]
Confocal laser scanning microscope	Expression patterns in aggrecan KO	WFA Hyaluronic acid HAPLP1 TnR Brevican	[26]
Confocal laser scanning microscope and epifluorescent microscope	Organization into geometrical patterns	WFA	[128]
Super-resolution structural illumination microscopy (SR-SIM)	Ultrastructure using topography measurements	WFA Aggrecan	[126]
Stochastic optical reconstruction microscopy (STORM)	Surface intensity and perforating synapses	WFA	[127]
